# Associations of maternal lipoprotein particle distribution in mid-pregnancy with birth outcomes: a pilot study

**DOI:** 10.1186/s12944-022-01664-4

**Published:** 2022-06-13

**Authors:** Todd C. Rideout, Xiaozhong Wen, Divya Choudhary, Marissa Catanzaro, Richard W. Browne, Vanessa M. Barnabei, Kai Ling Kong

**Affiliations:** 1 Department of Exercise and Nutrition Sciences, School of Public Health and Health Professions, University at Buffalo, Buffalo, NY USA; 2 Department Pediatrics, Jacobs School of Medicine and Biomedical Sciences, University at Buffalo, Buffalo, NY USA; 3 Department Biotechnical and Clinical Laboratory Sciences, Jacobs School of Medicine and Biomedical Sciences, University at Buffalo, Buffalo, NY USA; 4 Department Obstetrics and Gynecology, Jacobs School of Medicine and Biomedical Sciences, University at Buffalo, Buffalo, NY 14214 USA; 5 Baby Health Behavior Lab, Division of Health Services and Outcomes Research, Children’s Mercy Research Institute, Children’s Mercy Hospital, Kansas City, USA; 6 Department of Pediatrics, University of Missouri, Columbia, USA; 7 Center for Children’s Healthy Lifestyles and Nutrition, Medical Center, University of Kansas, Kansas City, KS USA

**Keywords:** Pregnancy, Lipids, Lipoproteins, Birth outcomes

## Abstract

**Background:**

An excessive rise in maternal lipids during pregnancy may have detrimental impacts on maternal and fetal health leading to adverse pregnancy outcomes. However, knowledge gaps exist with respect to the association between lipid biomarkers and birth outcomes.

**Methods:**

We conducted a secondary data analysis of healthy pregnant women (*N* = 25) with mid-pregnancy fasting serum samples collected at 22–28 weeks of gestation and birth outcome data. Serum was analyzed for conventional lipid profile (total-C, HDL-C, LDL-C, and triglycerides) and lipoprotein subclass distribution, including particle number (nM) and size (nm), for very low-density lipoprotein (VLDL)/chylomicron (CM), low density lipoprotein (LDL), and high-density lipoprotein (HDL), by nuclear magnetic resonance spectroscopy.

Associations between maternal lipids and birth outcomes, including birth weight (g) and gestational age (weeks), were assessed using multivariable linear regression, adjusted for pre-pregnancy BMI.

**Results:**

Although conventional lipids were not associated (*p* > 0.05) with birth outcomes, every 1-unit increment in large VLDL/CM particles (nM) and VLDL/CM size (nm) was associated with an increase in birth weight (confounder-adjusted β-coefficient, 45.80 g [5.30, 86.20, *p* = 0.003] and 24.90 g [8.80, 40.90, *p* = 0.002], respectively). Among the HDL subclass parameters, a 1-unit (nM) increase in the concentration of total HDL-particles was associated with a reduced birth weight (confounder adjusted β-coefficient, -19.40 g [95% confidence interval, -36.70, -2.20]; *p* = 0.03) after adjustment for maternal pre-pregnancy BMI.

**Conclusion:**

The preliminary results of this pilot study suggest that total particle concentrations of VLDL/CM and HDL in mid-pregnancy have divergent associations with birth weight, potentially reflecting the specific roles of these lipoprotein particles with respect to placental function and fetal growth.

## Background

To support placental development and optimal nutrient transfer to the growing fetus, a physiological rise in maternal blood lipids is observed during pregnancy, exemplified by transient but substantial increases in triglycerides (TG, ~ 200–400%), total-cholesterol (TC, ~ 25–50%), low-density lipoprotein cholesterol (LDL-C, ~ 70%), and high-density lipoprotein cholesterol (HDL-C, ~ 40%) [1, 2]. However, an excessive, non-physiological increase in maternal lipids during pregnancy has been reported to have detrimental effects on both maternal and fetal health. Excessive maternal hypertriglyceridemia during pregnancy, due to exacerbating secondary factors such as diabetes and obesity, has been associated with acute pancreatitis [3], preeclampsia [4], and adverse birth outcomes including preterm delivery and macrosomia [5]. Similarly, an excessive rise in maternal cholesterol during pregnancy, termed maternal hypercholesterolemia (MHC), has been associated with a range of pregnancy conditions including glucose intolerance [6], gestational diabetes [7], polycystic ovary syndrome [8], and preeclampsia [9].

As blood lipid assessment is not a routine component of obstetrical care [10], there are still knowledge gaps with respect to what constitutes a ‘normal’ blood lipid profile during pregnancy and the most appropriate lipid screening marker for maternal dyslipidemia. With numerous candidate biomarkers including standard lipid endpoints (i.e., TG, TC, LDL-C, HDL-C), apolipoproteins, as well as more advanced lipoprotein subclass distribution (i.e., particle number and size), there is little understanding of the biomarker(s) that is most reflective of an adverse maternal environment. Given these knowledge gaps, we conducted a pilot study to explore potential associations between pregnancy lipids, including conventional lipid panel and lipoprotein subclass distribution, and birth outcomes from a previous pregnancy cohort study.

## Methods

### Participants

This study was a secondary analyses of serum samples and birth outcome data collected as part of a previous pregnancy pilot cohort to examine the reinforcing value of high-energy-dense foods during pregnancy (not published). All procedures were approved by the Institutional Review Board at the University at Buffalo (STUDY00001381). The cohort consisted of 25, 18–35-year-old pregnant women with singleton gestations, recruited within the greater Buffalo, New York area between 2018–2019 through flyer distribution, social media posts, and local urban obstetrics clinics (Table 1). Women were excluded if they had a history of major chronic diseases (e.g., heart disease, diabetes) or were currently taking medications that affected appetite or insulin sensitivity.

**Table 1:** Characteristics of mothers and infants from the pilot cohort

Variable	Mean (SD)	n (%)	Range	
**Maternal Characteristics (*N* = 25)**				
Age (yrs)	30.7 (3.96)		21.6 – 37.8	
Race				
Caucasian		23 (92)		
African American		1 (4.0)		
Asian		1 (4)		
Education level (yrs)	16.1 (2.1)			
College graduate		23 (92)		
Pre-pregnancy BMI (kg/m^2^)				19.2–41.6
Normal		10 (40)		
Overweight		7 (28)		
Obese		8 (32)		
**Infant Characteristics**				
Male sex		16 (64)		
Birth weight (g)	3543 (333)			
Large for gestational age ^a^		10 (40)		
Gestational age at delivery (weeks)	39.28 (0.89)			

^a^ defined as birthweight greater than the 90th percentile for gestational age

### Data collection

Upon study enrollment, prospective women completed an interviewer-administered questionnaire to obtain information regarding general socio-demographics and reproductive and medical history. Maternal pre-pregnancy height and weight were collected by self-report at the initial visit and body mass index (BMI) was calculated as weight in kilograms divided by the square of height in meters. At mid-pregnancy between gestation weeks 22–28, a fasting blood sample was collected by a blood draw technician. A birth outcome questionnaire administered at a home visit within 7 days of birth was used to collect information on infant variables (i.e., date of delivery, sex, birth weight, and gestational age as assessed by ultrasound examination) and pregnancy complications (i.e., gestational diabetes, pre-eclampsia, and pre-term birth).

### Serum lipid analyses

Following a blood clotting period of 30 min at room temperature, maternal serum samples were separated by centrifugation at 3000 × g for 20 min and stored at -80 °C. TC, HDL-C, direct LDL-C, and TG were measured by direct automated enzymatic assay using diagnostic reagent kits, calibrators, and quality controls. Direct assessment of lipoprotein subclass distribution, including particle number (nM) and size (nm), for very low density lipoprotein (VLDL)/chylomicron (CM), low density lipoprotein (LDL), and high density lipoprotein (HDL), was conducted by nuclear magnetic resonance spectroscopy (NMR) using automated signal acquisition followed by computational analysis and proprietary signal processing algorithms (LabCorp Inc, Raleigh, NC) [11]. The diameter size ranges for each lipoprotein subclass were: VLDL/CM (large, > 60 nm; intermediate, 42–60 nm; small 29–41 nm); LDL (large, 23–29 nm; intermediate, 20.5–23 nm; small 18–20.5 nm) and; HDL (large, 9.4–14 nm; intermediate, 8.2–9.4 nm; small 7.3–8.2 nm). ApoB was measured by immunologic analyses (Liposcience, Raleigh, NC) and non-HDL cholesterol was calculated by subtracting HDL-C from TC.

### Statistical analyses

Associations between maternal second trimester lipids and continuous birth outcomes including birth weight (g) and gestational age (weeks) were assessed using multivariable linear regression. We considered maternal age and pre-pregnancy BMI as potential confounders that could affect both maternal pregnancy lipid status and birth outcomes. Significant confounders (*p* < 0.05), as estimated by linear regression, were included in the adjusted model. Two models were used in the analysis: (i) Model 1 was the crude model with second trimester lipid exposure only; and (ii) Model 2 adjusted for maternal pre-pregnancy BMI (the only significant confounder). All analyses were conducted using SAS version 9.4 (SAS Institute, Inc., Cary, NC). Statistical significance was set at a two-sided alpha level of *p* < 0.05.

## Results

Mothers included in this study were mainly Caucasian (92%), highly educated (92% college educated), and overweight or obese according to pre-pregnancy BMI (60%) (Table 1). With the exception of a breech birth (*n* = 1), no pregnancy complications were reported and mean infant birth weight and gestational age at delivery was 3543 ± 333 g and 39.28 ± 0.89 weeks, respectively.

Standard lipid profile measures, including cholesterol (TC, LDL-C, HDL-C, and non-HDL-cholesterol), TG, and apoB were unrelated (*p* > 0.05) to birth weight or gestational age (Table 2). After adjustment for maternal pre-pregnancy BMI, every 1-unit increment in large VLDL/CM particles (nM) and VLDL/CM size (nm) was associated with an increase in birth weight (confounder-adjusted β-coefficient, 45.80 g [95% confidence interval, 5.30, 86.20, *p* = 0.003] and 24.90 g [8.80, 40.90, *p* = 0.002], respectively) (Table 3). Although LDL subclass distribution was not associated with birth outcomes (Table 4), among the HDL subclass parameters, every 1-unit (nM) increment in the concentration of total HDL-particles was associated with a reduced birth weight (confounder-adjusted β-coefficient, -19.40 g [95% confidence interval, -36.70, -2.20]; *p* = 0.03) after adjustment for maternal pre-pregnancy BMI (Table 5). Lipoprotein subclass parameters for VLDL/CM, LDL, or HDL were not associated (*p* < 0.05) with gestational age.

**Table 2:** Association between maternal second trimester lipid profile and pregnancy outcomes

Lipid exposure	Birth weight (g) β (95% CI)^*^ *N* = 25	*P*-value	Gestational age (weeks) β (95% CI)^*^ *N* = 25	*P*-value
**Total-C (mg/dL)**				
Model 1	-0.80 (-4.20,2.60)	0.65	0.003 (-0.01,0.01)	0.57
Model 2	-0.40 (-3.80,3.00)	0.82	0.001 (-0.01,0.01)	0.81
**LDL-C (mg/dL)**				
Model 1	0.40 (-4.00,4.80)	0.86	0.002 (-0.01,0.01)	0.74
Model 2	0.70 (-3.60,5.00)	0.75	0.001 (-0.01,0.01)	0.88
**HDL-C (mg/dL)**				
Model 1	-4.90 (-11.10,1.20)	0.12	0.003 (-0.01,0.02)	0.73
Model 2	-4.20 (-10.60,2.30)	0.21	-0.002 (-0.02,0.02)	0.85
**nonHDL-C (mg/dL)**				
Model 1	0.80 (-3.20,4.80)	0.69	0.003 (-0.01,0.01)	0.65
Model 2	0.90 (-3.00,4.70)	0.66	0.002 (-0.01,0.01)	0.69
**Triglycerides (mg/dL)**				
Model 1	1.40 (-1.40,4.10)	0.32	0.003 (0.00,0.01)	0.45
Model 2	1.10 (-1.70,3.80)	0.45	0.005 (0.00,0.01)	0.21
**ApoB (mg/dL)**				
Model 1	1.30 (-4.90,7.50)	0.67	0.002 (-0.01,0.02)	0.78
Model 2	1.50 (-4.60,7.50)	0.63	0.002 (-0.01,0.02)	0.83

^*^Values are beta coefficients (95% CI) derived from linear regression models, representing the change in birth weight (g) or gestational age (weeks) with one unit increment in each lipid exposure; Model 1: crude model; Model 2: adjusted for maternal pre-pregnancy BMI

**Table 3:** Association between maternal second trimester VLDL/Chylomicron (CM) lipoprotein profile and pregnancy outcomes

Lipid exposure	Birth weight (g) β (95% CI)^*^ *N* = 25	*P*-value	Gestational age (weeks) β (95% CI)^*^ *N* = 25	*P*-value
**Total VLDL/CM particles (nM)**				
Model 1	-1.20 (-6.50,4.00)	0.64	-0.002 (-0.02,0.01)	0.80
Model 2	-1.60 (-6.70,3.50)	0.55	-0.001 (-0.01,0.01)	0.93
**Small VLDL/CM particles (nM)**				
Model 1	-3.40 (-11.20,4.40)	0.40	-0.014 (-0.03,0.01)	0.17
Model 2	-4.30 (-12.00,3.30)	0.27	-0.012 (-0.03,0.01)	0.25
**Medium VLDL/CM particles (nM)**				
Model 1	-2.10 (-12.50,8.40)	0.70	0.017 (-0.01,0.04)	0.24
Model 2	-1.40 (-11.70,9.00)	0.80	0.014 (-0.01,0.04)	0.30
**Large VLDL/CM particles (nM)**				
Model 1	**48.30 (10.70,85.80)**	**0.01**	0.026 (-0.09,0.14)	0.65
Model 2	**45.80 (5.30,86.20)**	**0.03**	0.070 (-0.04,0.18)	0.22
**VLDL/CM size (nm)**				
Model 1	**25.90 (9.80,42.00)**	**0.002**	0.023 (-0.03,0.07)	0.30
Model 2	**24.90 (8.80,40.90)**	**0.002**	0.029 (-0.02,0.08)	0.23

^*^Values are beta coefficients (95% CI) derived from linear regression models, representing the change in birth weight (g) or gestational age (weeks) with one increment change in each lipid exposure; Model 1: crude model; Model 2: adjusted for maternal pre-pregnancy BMI

**Table 4:** Association between maternal second trimester LDL lipoprotein profile and pregnancy outcomes

Lipid exposure	Birth weight (g) β (95% CI)^*^ *N* = 25	*P*-value	Gestational age (weeks) β (95% CI)^*^ *N* = 25	*P*-value
**Total LDL particles (nM)**				
Model 1	0.20 (-0.30,0.70)	0.42	0.00 (0.00,0.00)	0.70
Model 2	0.20 (-0.20,0.70)	0.37	0.00 (0.00,0.00)	0.76
**Small LDL particles (nM)**				
Model 1	0.00 (-0.50,0.50)	0.93	-0.001 (0.00,0.00)	0.11
Model 2	-0.10 (-0.60,0.40)	0.64	-0.001 (0.00,0.00)	0.24
**Intermediate LDL particles (nM)**				
Model 1	0.30 (-0.50,1.10)	0.47	0.002 (0.00,0.00)	0.09
Model 2	0.30 (-0.50,1.10)	0.44	0.002 (0.00,0.00)	0.08
**Large LDL particles (nM)**				
Model 1	0.10 (-0.30,0.50)	0.66	0.001 (0.00,0.00)	0.37
Model 2	0.20 (-0.20,0.70)	0.38	0.001 (0.00,0.00)	0.67
**LDL size (nm)**				
Model 1	-141.50 (-386.80,103.80)	0.26	0.466 (-0.18,1.11)	0.16
Model 2	-111.80 (-362.10,138.60)	0.38	0.348 (-0.30,1.00)	0.20

^*^Values are beta coefficients (95% CI) derived from linear regression models, representing the change in birth weight (g) or gestational age (weeks) with one unit increment in each lipid exposure; Model 1: crude model; Model 2: adjusted for maternal pre-pregnancy BMI

**Table 5:** Association between maternal second trimester HDL lipoprotein profile and pregnancy outcomes

Lipid exposure	Birth weight (g) β (95% CI)^*^ *N* = 25	*P*-value	Gestational age (weeks) β (95% CI)^*^ *N* = 25	*P*-value
**Total HDL particles (μM)**				
Model 1	**-20.90 (-37.80,-3.90)**	**0.02**	-0.009 (-0.06,0.05)	0.75
Model 2	**-19.40 (-36.70,-2.20)**	**0.03**	-0.018 (-0.07,0.03)	0.51
**Small HDL particles (μM)**				
Model 1	2.60 (-24.20,29.50)	0.85	-0.033 (-0.10,0.04)	0.36
Model 2	1.00 (-25.30,27.40)	0.94	-0.027 (-0.09,0.04)	0.43
**Medium HDL particles (**μ**M)**				
Model 1	-14.30 (-34.50,6.00)	0.17	0.003 (-0.06,0.05)	0.93
Model 2	-12.80 (-32.90,7.30)	0.21	-0.001 (-0.06,0.04)	0.73
**Large HDL particles (μM)**				
Model 1	-19.60 (-56.80,17.50)	0.30	0.044 (-0.06,0.14)	0.39
Model 2	-15.50 (-53.10,22.00)	0.42	0.027 (-0.07,0.13)	0.59
**HDL size (nm)**				
Model 1	55.80 (-361.80,473.30)	0.79	0.923 (-0.13,1.98)	0.09
Model 2	107.60 (-306.40,521.60)	0.61	0.773 (-0.27,1.81)	0.14

^*^Values are beta coefficients (95% CI) derived from linear regression models, representing the change in birth weight (g) or gestational age (weeks) with one unit increment in each lipid exposure; Model 1: crude model; Model 2: adjusted for maternal pre-pregnancy BMI

## Discussion

The results of this pilot study suggest that specific maternal lipoprotein subclass parameters in pregnancy may be predictive biomarkers of infant birth weight. Although birth weight was not associated with conventional lipid assessments, unit increases in the concentration of mid-pregnancy VLDL/CM lipoproteins (total particle concentration and size) were associated with increased infant birth weight while total HDL particles were associated with reduced birth weight. This divergent lipoprotein-specific association may reflect the unique functional roles of VLDL and HDL lipoproteins in maternal fuel metabolism and placental/fetal development during pregnancy.

Pregnancy is associated with adaptive changes in the metabolism of the large triglyceride-rich lipoproteins including chylomicrons of intestinal origin and VLDL which transport de novo synthesized lipids from the liver. By late pregnancy, serum TG increases by 200–400% due to a decrease in insulin sensitivity. The subsequent influx in adipose-derived fatty acids to the liver results in an estrogen-induced increase in VLDL production and secretion [2]. Late pregnancy is further associated with reduced clearance of TG-rich lipoproteins due to a decrease in lipoprotein and hepatic lipase [12]. Maternal–fetal transfer of fatty acids is mediated by lipoprotein receptor binding and extracellular TG hydrolysis by lipase expression on the placental syncytiotrophoblast layer [13]. The association we observed between mid-pregnancy VLDL/CM (total particle concentration and size) and birth weight is consistent with the role of TG-rich lipoproteins in transporting fatty acid fuel for placental uptake. However, maternal TG concentration was not associated with birth weight, even though previous studies have reported a positive association [14–16]. Only limited work has examined potential associations between VLDL subclass distribution and fetal growth parameters. VLDL particle concentration in mid-pregnancy has been reported to be increased in women with gestational diabetes [17], a pregnancy condition often linked to large for gestation age birth [18]. However, the finding of Roland et al. [19] suggest that low placental CETP activity in late gestation was associated with reduced TG exchange, large TG-rich VLDL/chylomicron particles, and reduced fetal growth, potentially reflecting altered placental lipid uptake.

Interestingly, neither maternal TG nor VLDL subclass parameters in mid-pregnancy were associated with gestation age. Previous work has reported that elevated maternal TG concentration in pre-pregnancy [20] and early pregnancy (< 15 weeks) [21] were associated with an increased risk of pre-term birth, a finding that is somewhat supported by a previous systematic review, although data between studies were highly heterogeneous [22]. Further, Catov et al. (2017) reported that low total VLDL particle concentration (mainly due to fewer small VLDL particles) in the first trimester was associated with pre-term birth [23]. This data might suggest that early pregnancy TG/VLDL status might be more predictive of pre-term birth than mid-pregnancy concentrations, however, future work should examine how the trajectory of changes in lipid and lipoprotein concentrations across pregnancy influence gestation age.

Although total and LDL-C concentrations increase substantially throughout gestation, the rise in maternal HDL-C is relatively minor and has been reported to peak in the 2^nd^ trimester [24, 25]. There is a dearth of research pertaining to HDL subclass distribution during pregnancy, however, a shift in HDL subclass particles is thought to occur throughout the gestation period. Previous studies have reported an increase in larger, TG-rich HDL particles (HDL2a) and a reduction in smaller (HDL3a and b) species throughout gestation [26–28]. Alternatively, Zeljkovic et al. (2013) [24] reported an increase in small HDL particles (HDL3b and 3c) and a concurrent reduction in small HDL2a particles as pregnancy progressed. These conflicting reports may reflect the complex array of metabolic factors regulating HDL remodeling including plasma factors, adiposity status, and yet-to-be identified pregnancy-induced factors [29, 30].

Outside of pregnancy, HDL is most typically recognized as a cardioprotective mediator of reverse cholesterol transport. However, during pregnancy, HDL has a range of other diverse functions that may specifically influence placental metabolism and fetal growth, as recently reviewed by Woollett et al. [31]. First, as a lipid carrier, HDL serves to directly support fetal growth by delivering lipid fuels through placental lipoprotein receptors and perhaps indirectly by sparing of maternal glucose for placental uptake [32]. Second, a diverse range of bioactive proteins are associated with specific HDL subclasses including apolipoprotiens, enzymes, and complement proteins, which confer antioxidative, anti-inflammatory, and immune functions to HDL [33]. The recently observed pregnancy-specific shift in the HDL proteome suggests critical, but as yet unknown, role(s) for specific HDL subclasses in fetal growth and development [28]. For instance, an increase in the proportion of larger HDL particles in pregnancy has previously been associated with preterm birth [23] and reduced birth length and head circumference [24]. Interestingly, in our cohort, we observed a negative association between total HDL particles and birth weight, but a similar association with HDL-C was not observed, highlighting a potentially important function of HDL in pregnancy beyond cholesterol transport. In support of this finding, a recent study reported that women giving birth to small-for-gestational age (SGA) infants had higher total HDL particles at 36–38 weeks of pregnancy compared with women giving birth to appropriate-for-gestational age (AGA) infants [19]. Similarly, Kramer et al. previously reported an elevated risk of SGA infants across quartiles of maternal total (adjusted odds ratio, 2.8; 95% CI, 1.7–4.5) and small (1.6; 1.04–2.5) HDL particle concentration between 24–26 weeks of gestation. As some studies have reported a protective effect of HDL-C against low birth weight [34], the authors suggested that this counterintuitive finding was not likely causative but rather a consequence of placental dysfunction. Likewise, while our findings may reflect a dysregulation in HDL particle turnover, a more direct causative influence of HDL particles on fetal growth cannot be ruled out given the emerging recognition of complex changes in HDL subclass structure and function during pregnancy and their relationship with birth outcomes.

## Conclusions

In conclusion, the preliminary results of this pilot study suggest that total particle concentrations of VLDL/CM and HDL in mid-pregnancy have divergent associations with birth weight, potentially reflecting the specific roles of these lipoprotein particles with respect to placental function and fetal growth. However, our findings must be interpreted with caution given several limitations including a small sample size potentially reflecting a type-1 statistical error, lack of statistical power to examine effect modification by infant sex, collection of birth outcome data with a maternal-reported birth outcome questionnaire, restriction of our analyses to one mid-pregnancy timepoint, and a lack of diversity with respect to race/ethnicity and education. Despite these limitations, our findings can inform future work to examine how the trajectory of lipoprotein distribution across pregnancy influences birth outcomes in a larger and more diverse longitudinal cohort.

## Data Availability

The datasets used and/or analysed during the current study are available from the corresponding author on reasonable request.

## Abbreviations

TG: Triglycerides
TC: Total-cholesterol
LDL-C: Low-density lipoprotein cholesterol
HDL-C: High-density lipoprotein cholesterol
VLVL/CM: Very low density lipoprotein/chylomicron
NMR: Nuclear magnetic resonance spectroscopy
LDL: Low density lipoprotein
HDL: High density lipoprotein
MHC: Maternal hypercholesterolemia
BMI: Body mass index
AGA: Appropriate-for-gestational age
SGA: Small-for-gestational age
